# A Dynamic Model of Evolutionary Knowledge and Capabilities Based on Human-Machine Interaction in Smart Manufactures

**DOI:** 10.1155/2022/8584888

**Published:** 2022-04-26

**Authors:** Shuxian Chen, Zongqiang Ren, Xikai Yu, Ao Huang

**Affiliations:** School of Business, Wenzhou University, Wenzhou 325035, China

## Abstract

The increasing use of smart machines and devices is not only changing production principles but also reshaping the value of cocreation logic. The interaction between human and smart machine is the enabler of generating augmented intelligence. A system dynamics model is abstracted from smart manufacturing practices to represent the evolutionary processes of inertia, capability, and reliability induced by human-machine interaction. Human-machine interaction is conceptualized into two dimensions: technical and cognitive interaction. Simulation experiments illustrate how the improvement of human-machine interaction can leverage the dynamic capability and reduce the inertia in enterprises through multiple nonlinear feedbacks. There are two pathways to improve reliability and performance in enterprises by human-machine interaction: (1) to promote initiative innovation (change) from endogenous enabler by improving dynamic capability and (2) to promote transformation of knowledge and variation triggered by exogenous environmental changes to improve the dynamic capability for the flexibility and reliability.

## 1. Introduction

Modern technologies are redefining and reshaping lifestyles and social and economic practices, especially machines embedded with intelligence can do complex tasks and even generate art and new knowledge, not just simple, routinized tasks. Smart manufacturing is not only a production mode in Industry 4.0 but also a new value creation logic and innovation paradigm for the new industrial revolution [1, 2], for example, it reshaped the command and control paradigm in traditional management theory [3]. Organizational innovation in this digital transformation landscape has become a fast-growing and highly important arena for research by understanding the implications of these trends to inform designing for social and economic change.

Given the soaring numbers of smart machines used in workplaces, they are becoming our new colleagues to cocreate value with and for their users [4, 5]. Intelligent organizations in future need to blend technology-enabled insights of smart machines with a sophisticated understanding of human emotion-cognition. Accordingly, cooperation and interaction of humans with smart machines for augmented intelligence are becoming more important than ever. It is time to consider the strategic role of smart machines, new colleagues of human beings, for organizational change. Numerous literatures have investigated the complex relationships, between human and machine, such as technical frameworks, social norms, and networking principles, even emerging a new discipline of ethorobotics [6]. Nonetheless, most of them focused on socio-technical, psychological, and behavioral problems from micro (individual) and macro (social) levels. These conclusions have been exercised at a meso level (organizational) and are little examined. Theoretically, it remains unclear how human-machine interactions shape or enable the change of an organization, in instances such as structural evolution, capability transformation, or production innovation.

This paper explores the mechanism capability transformation enabled by human-machine interaction in the workplace by system dynamic model, which are

1. Identifying and representing dynamic elements related to dynamic capability for the smart manufacturing.
2. Investigating the evolution of knowledge, inertia, capability, and variation in the human-machine interaction to promote the performance.

## 2. The Ories and Models

### 2.1. Human-Machine Cooperation: Technical and Cognitive Interaction

Human-machine interaction is defined as the interaction and communication between human users and machines through multiple interface channels in a dynamic environment [1]. Scholars summarized the evolution of human-machine interaction based on the developing functions of machines [7, 8]. In early work, machines were operated as tools to improve productivity and quality. With the development of electrification and automation, machines can facilitate taskwork by automating production. After the 1970s, with the introduction of information and communication technology, machines could leverage people's physical and cognitive (e.g., data retrieval and processing) capabilities on the centrality of humans [9]. In the latest industrial revolution (Industry 4.0), the extensive application of digital technologies is promoting the interaction between workers and machines to a new level of interaction of heterogenic agents [10], network communication, cognitive processes, and emotional bounding which are emerging in human-machine interaction systematically.

From the evolution of the relationship between man and machine, we can grasp that the interaction between man and machine evolves along technical and cognitive dimensions. Technical dimension refers to the changes in the way and content of human-machine interaction brought by technology [11]. For example, technologies like the Internet of things (IOT), artificial intelligence (AI), and cloud computing promote multichannel interaction between humans and machines throughout a highly networked environment [2, 12]. Schoemaker and Tetlock [13] argued that the most intelligent organizations will need to blend technology-enabled insights with a sophisticated understanding of human reasoning and creativity in the coming years. Today's robotics become more powerful, more flexible, and smarter. Ultimately, these robots will not only be able to communicate with each other, but it is more safe and smarter to work with and even learn new skills from humans.

The cognitive dimension refers to the changes in the social and psychological relations in human-machine interacting as peers or companions, such as trust, social recognition, and emotional-cognitive creativity [11, 14, 15]. Ethological approach shows that social robotics can provide a more plausible functional human-robot interaction [16]. For example, humans are willing to accept robots as trustable partners if they can ascribe some form of awareness and true understanding to them [16, 17]. Cognitive dimension of human-machine interaction will define the partnerships as the centrality of human-machine in the future, which means that the coexistence of humans and machines can make both sides smarter over time [6].

### 2.2. Human-Machine Interaction: Enabler for Development

Organizations are increasingly engaging in digital competition that is enabled or induced by information technology [18]. To create a more intelligent enterprise, executives need to leverage the strengths of both humans and computers in order to produce superior judgments [13]. Intelligent machines as coworkers, rather than technological equipment, may result in a profound transformation to organization, from the strategy structure to the behaviors and feelings of the workers [17, 19]. Furthermost, the cooperation between smart machines and humans generates augmented intelligence, allowing them to become more attuned to the competitive dynamics to integrate, establish interaction with internal and external resources, and deal with environmental changes [13, 20]. These distinctive processes including ways of coordinating and combining are the base of capability generation in smart manufacturing.

Firstly, inertia is institutionalized into a regulatory structure to shape (be shaped by) people's behavior in an organization [21]. Intelligent machine changes the formation and existence of inertia plus routine of organization around which organizations are constructed also through which they operate. For example, ERP can replace the traditional manual procurement and logistics management processes with procedures. Knowledge is the basic material to produce capabilities; at the same time, some capabilities will also be solidified as inertia in the organization by means of stocked knowledge. However, in today's turbulent market environments, firms are increasingly facing challenges to keep their knowledge base up-to-date and upgrade their dynamic capabilities [22].

Secondly, human-machine interaction not only improves the ability of organization but also expands the sourcing and way for ability and knowledge generation [23]. Organizational capability is no longer depended on what humans do well or poorly [5]. Organizational capabilities are defined as a body of knowledge about how to do things [24]. On the other hand, the smart machine can also produce knowledge by itself and complement human capabilities to form augmented intelligence. Additionally, smart machines just like people become the dualistic carriers of capabilities and strategic advantages will increasingly depend on a shared capacity of human and machine [13, 25].

Thirdly, according to evolutionary theories, the innovative agents are evolving into the dualistic actors of human and machine which would trigger more variation. For example, networking machines can expand the scope of knowledge search and speed up information processing. Successful variations are retained and turned into knowledge or capabilities that can be invoked when needed to improve the performance. Performance is measured in the desired levels of reliability to respond flexibly to changes.

**Figure 1:**
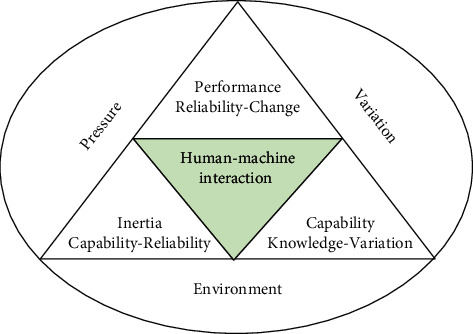
Research framework.

In summary, we integrate the key dynamic elements related to dynamic capability based on the human-machine interaction to investigate the inertia accumulation, capability formation, and the reliability growth in the environment turbulence (Figure 1). This framework includes internal and external changes across the boundary of organization: external changes mainly reflect the triggers of environmental changes on organizational adaptability, internal changes demonstrate the interdependence and feedbacks of variables within organizations to generate capabilities for adaptability.

## 3. Model Structure and Methods

Drawing on organizational inertia and capabilities dynamic accumulation model proposed by Larsen and Lomi [24], we construct a dynamic model to capture the complex relationship among the capability, inertia, knowledge, and variation based on human-machine interaction. System dynamic modelling has specific advantages to investigate complex dynamic relationships between variables in multiple feedbacks by the observation-generating mechanism [24, 25, 26]. For example, the real-time data tracking and sensemaking capabilities of intelligent technology provide context-aware and curated information to users, enabling them to generate dictate precisely decisions and control the directions of variations. Our strategy is to keep the notation as much intuitive as possible with the practice of smart manufacturing in enterprise. Accordingly, we hypothesize the microstructural relations of components with each model equation from empirical studies and field practice.

### 3.1. Organizational Inertia

Enterprises' inertia generates from organizational routines and experience as well as culture in business contexts, which is social construal process based on knowledge production, accumulation, and usage. Knowledge can be measured by a stock-and-flow approach [27]. Structural inertia (I) in an enterprise is defined as a stock (or accumulator) variable that integrates the corresponding net flow between an increase in inertia I (+) and a decrease in inertia I (−).

(1) $$\begin{array}{c} I_{t}=\int_{t_{0}}^{t}\left[I^{\left(+\right)}\left(s\right)-I^{\left(-\right)}\left(s\right)\right]\text{d}s+I\left(t_{0}\right). \end{array}$$

Inertia accumulates over time and is affected by enterprise size (S), accumulation (inertia), and experience (capability from practice). To represent these concepts, we specify logistic functional relations between increase in inertia, size, and capabilities. Furthermore, we assume that inertia will increase by a small amount of capability every year at the rate of *μ*,

(2) $$\begin{array}{c} \frac{\text{d}\left(I^{+}\right)}{\text{d}t}=s*Ln\left(I_{t}-C_{t}\right)*Ln\left(C_{t}\right)+\mu *Ln\left(C_{t}\right). \end{array}$$

We assume that change attempts (CA) are intendedly adaptive and have the basic objective of decreasing structural inertia, which means the innovation will decrease the inertia in the environment turbulence. So, we model the decrease in inertia as a random variable which represents how much inertia decreases when a change attempt is initiated in the environment turbulence (E). *E* is the stochastic variable determining the actual level of environmental turbulence.

(3) $$\begin{array}{c} I_{t}^{\left(-\right)}=\left\{\;\begin{array}{c} 0,\;\text{if }{\text{CA}}_{t}\leq 0,\\ 0.1*I_{t}*E,\;\text{if }{\text{CA}}_{t}>0. \end{array}\right. \end{array}$$

### 3.2. Change Pressure and Change Attempts

Change attempts (CA) are triggered by the accumulation of the pressure for change (PC) in environmental turbulence. When the pressure for change becomes bigger than the actual threshold (AT), change attempts emerge (i.e., CA takes on a value of 1). The actual threshold for change (AT) is a function of a baseline threshold (BT) and environmental turbulence (E) at the level of inertia in the enterprise. Therefore,

(4) $$\begin{array}{c} {\text{CA}}_{t}=\text{IF THEN ELSE }\left({\text{CA}}_{t}\geq 0.1,{\text{PC}}_{t}\times \zeta ,0\right),\\ {\text{AT}}_{t}=\left\{\;\begin{array}{c} 0,\;\text{if }E<\text{BT,}\\ e*Ln\left(I_{t}\right),\;\text{if }E\geq \text{BT}, \end{array}\right. \end{array}$$

where the BT can be interpreted as the minimum level that pressure for change (PC) must reach in order to trigger change attempts, and *e* is the average value in the random function of environmental turbulence.

Pressure for change cumulates over time as the actual level of performance diverges from the expected level of performance expressed in terms of reliability. Change attempts then diminish the pressure for change that is represented as

(5) $$\begin{array}{c} {\text{PC}}_{t}=\int_{t_{0}}^{t}\left[{\text{PC}}^{\left(+\right)}\left(s\right)-{\text{PC}}^{\left(-\right)}\left(s\right)\right]\text{d}s+\text{PC}\left(t_{0}\right). \end{array}$$

The pressure for change increases is defined as a function of the gap between expected reliability (ER) and reliability (R) which will accumulate into additional units of pressure for change. However, if actual reliability is better than expected, then no additional pressure for change is recorded. The Max operator is used for this function as follows:

(6) $$\begin{array}{c} {\text{PC}}_{t}^{\left(+\right)}=\text{Max}\left(0,{\text{ER}}_{t}-R_{t}\right). \end{array}$$

If change attempts are made, the pressure for change will decrease. The actual effect of this decrease is based on how successful the change attempt is, so the random component *ζ* is included in the equation that regulates the decrease in pressure for change.

(7) $$\begin{array}{c} {\text{PC}}_{t}^{\left(-\right)}=\left\{\begin{array}{c} 0,\;\text{if }{\text{CA}}_{t}\leq 0,\\ {\text{PC}}_{t}*\zeta ,\;\text{if }{\text{CA}}_{t}>0. \end{array}\right. \end{array}$$

### 3.3. Performance and Reliability

Reliability as an indicator of performance is the joint consequence of routinization, formalization, and institutionalization in an organization. So, the increase in reliability is based on the capability, which is enhanced by man-machine interaction (coHM). Increase in reliability in the baseline variability is given as

(8) $$\begin{array}{c} R_{t}^{\left(+\right)}=v_{t}-R_{t}\\ =\text{BV}*C_{t}*coHM-R_{t}. \end{array}$$

The decrease in reliability is defined as a threshold function in the same spirit of above I (−) and PC (−), which means any change attempt would decrease the reliability. The expected reliability (ER) will depend on a trend observed from previous periods and on an explicit managerial goal at the specific level of reliability. TR is the trend in reliability, and SR is the “stretch” in reliability (strategic goal). The trend in reliability is formulated as a first-order exponential smoothing of reliability pushed by the development of human-machine technology. The stretch of reliability as a proxy for the expectations of a steady increase in performance from period to period may be influenced by several managerial factors and defined as a fractional improvement over the level of reliability.

(9) $$\begin{array}{c} {\text{ER}}_{t}=R_{t}+{\text{TR}}_{t}+{\text{SR}}_{t},\\ \text{TR}=\text{Smooth}\left(\;R_{t}\cdot \frac{R_{t}-{\text{AR}}_{t}}{{\text{AR}}_{t}},\tau \right)*Ln\left(te\text{HM}+1\right). \end{array}$$

### 3.4. Capability, New Knowledge, and Variation

The cognitive interaction of human-machine interaction moderates the increase in capabilities generated by new knowledge and the transformation process. The decrease of capabilities comes from change attempts, which means the existing capability is outdated. This function can be formalized in the following way:

(10) $$\begin{array}{c} C_{t}=\int_{t_{0}}^{t}\left[\text{LR}\left(s\right)-\text{EC}\left(s\right)\right]\text{d}s+C\left(t_{0}\right), \end{array}$$

where the LR is the learning rate which is affected by the cognitive interaction of human and machines. A high level of cognitive interaction can enhance the quality of transformation from knowledge to capability.

(11) $$\begin{array}{c} {\text{LR}}_{t}={\left(1+co\text{HM}\right)}^{2}*Ln\left({\text{NK}}_{t}+1\right). \end{array}$$

New knowledge (NK) mainly generates from two sources: variations and cocreation of human-machine. According to evolution theory, some variations will be selected and converted into new knowledge retained in organizations, which can be leveraged by the synergy of human-machine interaction. At the same time, the synergy of human-machine will create new knowledge. For example, people can get new insights from machine operation by machine-learning.

(12) $$\begin{array}{c} {\text{NK}}_{t}=\int_{t_{0}}^{t}\left[\text{RR}\left(s\right)-\text{LR}\left(s\right)\right]\text{d}s+\text{NR}\left(t_{0}\right). \end{array}$$

The retention rate (RR) is in two parts: the selected fraction of variation that is leveraged by the synergy of human-machine and the new knowledge that is cocreated by human-machine.

(13) $$\begin{array}{c} {\text{RR}}_{t}=Ln\left(Vs*V_{t}+1\right)+{\left(1+coHM\right)}^{2}, \end{array}$$

where VS is the selected variation to be retained in the enterprise, which is defined by the technical interaction and cognitive interaction of human-machine. The technology interaction of human-machine can diminish the variability in management, thus decreasing the number of variations to be selected for retention, such as precise decision by big data so that

(14) $$\begin{array}{c} {\text{VS}}_{t}=\frac{\cdot \left(\text{coHM}+te\text{HM}\right)}{1+te\text{HM}*so\text{HM}}. \end{array}$$

Variation (V) originated from the change attempts and innovations in the environment turbulence based on previous reliability. So, the formulation is described as

(15) $$\begin{array}{c} V_{t}=\int_{t_{0}}^{t}\left[\text{EP}\left(s\right)-\text{VSO}\left(s\right)\right]\text{d}s+V\left(t_{0}\right), \end{array}$$

where EP is the amount of exploration going on in one time period and VSO is the variation selected out, i.e., not adopted by the enterprise, which is a fraction of the total variation minus the selected variation. The exploration is based on the average reliability of enterprises to meet the change attempts of environmental turbulence and is described as

(16) $$\begin{array}{c} {\text{EP}}_{t}={\text{CA}}_{t}*{\text{EN}}_{t}+Ln\left({\text{AR}}_{t}+1\right)*\left(1+\text{teHM}\right). \end{array}$$

**Figure 2:**
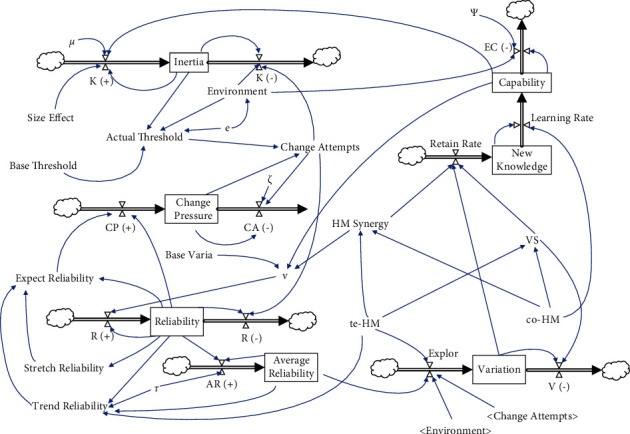
Multiple feedback for inertia, capability, variation, and reliability.

The holistic model is presented in Figure 2. The quality and validity of second-order models are tested by internal validity and correspondence between a theoretical narrative and its reconstruction [24].

## 4. Simulation Result and Analysis

The key enablers in this model are the environments turbulence, technological interaction of human and machines, and cognitive interaction of human and machines. Environment turbulence is defined as a stochastic variable which obeys the random normal distribution (mean = 5, S.D. = *e*), and *e* is an indicator to reflect the average degree of environmental change. Technological interaction (teHM) and cognitive interaction (coHM) are evaluated based on human-machine evaluation criteria ranging from 0 to 1. Cognitive interaction includes emotional and cognitive aspects, such as friendly interfaces, comfortable feelings, and situated learning. Technological interaction includes function, flexibility, and efficiency, such as visibility of system status, timely response, and smart control.

The simulation results are modeled by the software Vensim•PLE.8.1.0 with numerical integration using the fourth order Runge–Kutta method in a fixed step. This software for SD simulation can be used to define the stochastic components of the models via internal embedded random-number generation in this model, such as environmental turbulence or pressure regulation. Below, we present the simulation results under different scenarios.

### 4.1. Scenario 1 (Baseline Model)

**Figure 3:**
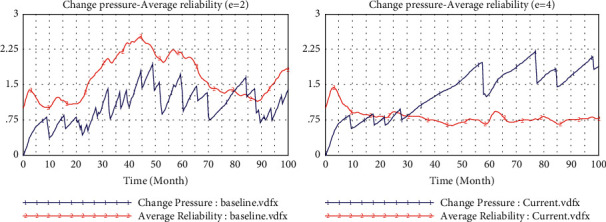
Comparison of environmental changes.

**Figure 4:**
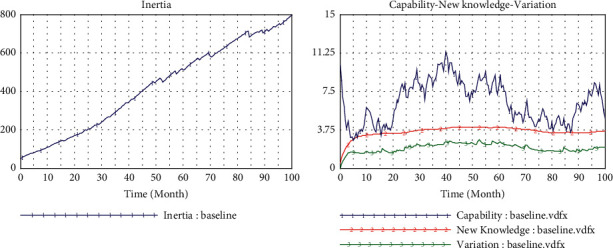
Baseline model.

Figure 2 shows the simulation results of the baseline model, which operates at a low level of technological interaction (teHM = 0.2) and cognitive interaction (coHM = 0.2) of human-machine in the low level environment turbulence (*e* = 2).  Figure 3 presents the comparison of operation results of environmental changes (from *e* = 2 to *e* = 4). In Figure 4, we can observe that inertia is building up over time which will accumulate and convert into organizational rigidity. However, the dynamic capability of enterprises only fluctuates at a low level based on the associated new knowledge and variation.

When the environment turbulence increases (*e* from 2 to 4) resulted in the bigger pressure for change (Figure 4), reliability has not increased but even decreased accordingly, which means that enterprises who just rely on their inertia without dynamic capability cannot respond to environmental changes timely. In this condition, according to these multiple causal cycles, the dynamic capability cannot provide strong support for enterprises to meet the demand of change attempts in the environment of turbulence. Thus, it is impossible to produce enough variation and new knowledge in the main drivers of capabilities.

### 4.2. Scenario 2 (teHM = 0.2, coHM = 0.6)

**Figure 5:**
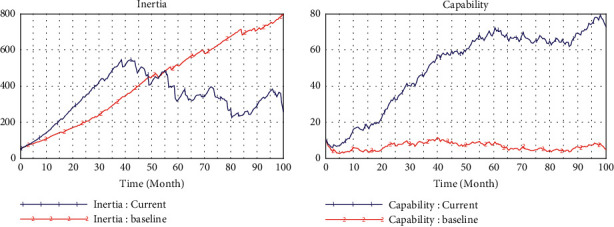
Simulation in high level cognitive interaction (0.6).

**Figure 6:**
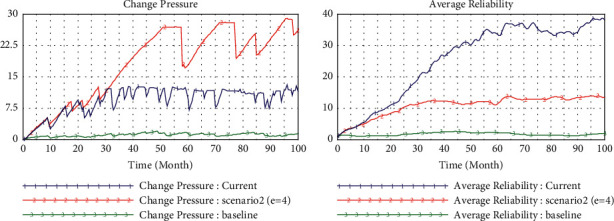
Comparison of environmental changes.

To probe further the enabler of human-machine interaction for inertia and capabilities, in scenario 2, we improve the level of human-machine cognitive interaction step by step (from 0.2 to 0.6) and set the level of human-machine technological interaction as constant (teHM = 0.2). We find that the inertia will increase with the incremental level of cognitive interaction, but as the level of cognitive interaction exceeds the average value (0.5), the accumulation of inertia will decrease after experiencing rapid growth in the initial stage (Figure 5). In this case, the dynamic capability has been growing steadily at a high level. The most fascinating finding is that the change pressure increases dramatically at the constant indicator of the environment (*e* = 2), which is triggered by the endogenous enabler (capability) (Figure 6). Furthermore, the enterprise reliability is still growing greatly, and this growth mainly comes from dynamic capability rather than inertia. So, cognitive interaction of humans and machines can improve the dynamic capability to generate growth and reduce organizational rigidity.

### 4.3. Scenario 3 (teHM = 0.6, coHM = 0.2)

**Figure 7:**
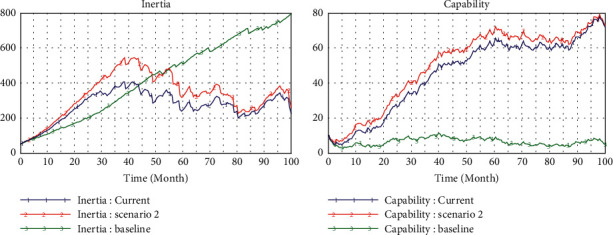
Simulation in high level of technical interaction (0.6).

By the same reasoning above, we improve the level of technical interaction step by step (from 0.2 to 0.6) to examine the effects of human-machine technological interaction. The evolutionary trajectory of inertia is the same as that in scenario 2, but it has more lag in time (Figure 7). Although the amount of variation and new knowledge induced by innovation is greater than that of scenario 2, the dynamic capability is lower than that of scenario 2, which may be due to the low support of human-machine cognitive interaction for knowledge transformation and learning efficiency.

Compared with scenario 2, under the same conditions, the effect of improving technical interaction of human-machine on capability is approximately the same as that of improving cognitive interaction, but it can effectively reduce the organizational inertia than latter.

### 4.4. Scenario 4 (teHM = 0.5, coHM = 0.5, *e* = 2)

**Figure 8:**
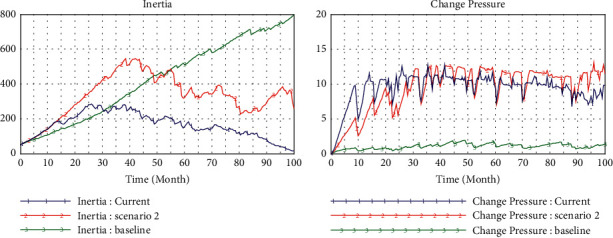
Simulation in medium level cognitive and technical interaction (0.5).

**Figure 9:**
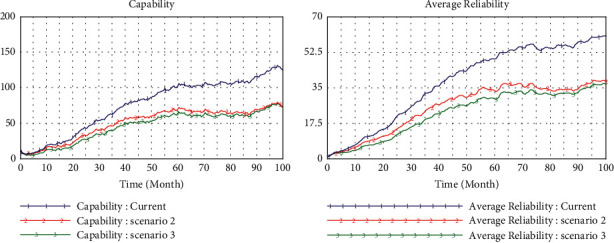
Comparison in different scenarios.

Both cognitive interactions and technical interactions between humans and machines are improved to a medium level of 0.5 in scenario 4. In Figure 8, we observe the level of inertia accumulation is lower than that in scenario 2, and the change pressure is almost the same level with that of scenario 2 and scenario 3 at the same environment turbulence (*e* = 2). However, in Figure 9, the evolutionary trajectory of capability and reliability is almost the same outline with that in scenario 2 and scenario 3, but whose value is significantly greater than the latter two. This is an interesting revelation because it means we can achieve the same result by improving one dimension of human-machine interaction significantly or balancing two dimensions to a medium level.

### 4.5. Scenario 5 (teHM = 0.9, coHM = 0.9, *e* = 4)

**Figure 10:**
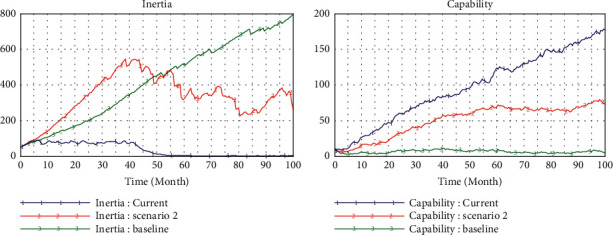
Simulation in high level of cognitive and technical interaction (0.9).

**Figure 11:**
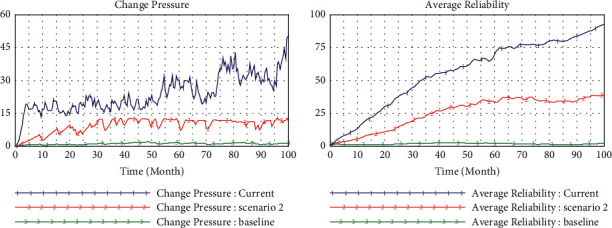
Comparison in different scenarios.

In scenario 5, we simulate the relationship between inertia and dynamic capability when human-machine interaction reaches a perfect level in violent environmental turbulence (*e* = 4). The result shows that the accumulation of inertia remains at a very low level and almost disappears (Figure 10). At this stage, enterprises will become capability-driven organizations where capability is embedded in the human-computer interactive systems of organizations which inspire more attempts for change (Figure 11). Accordingly, enterprises can smartly respond to environmental changes through ingenious human-machine cooperation without the constraints of inertia and rigidity.

From the simulating results above, we can find the cognitive and technical interaction of human-machine can affect the evolution trajectory of inertia, capability, and knowledge, even the variation in organizations. If organizational inertia are solidified into organizational systems and process specifications, they will be transformed into organizational rigidity, which can not only maintain the stability of the organization but also hinder the adaptability of the organization to the environment. So, dynamic capability is taken as knowledge reconfiguration capability [20]. For example, the cognitive interaction of human-machine can promote the new knowledge convert to the internal capital as form of capability, and the technical interaction of human-machine can promote variation by the dynamically exchanged with external players.

## 5. Discussion and Implications

Based on smart manufacturing in enterprises, we present an abstract model of operations systems as representing the evolution of inertia, capability, and innovation induced by human-machine interaction in the environment of turbulence and some of the assumptions and propositions in system dynamics terms. The simulations show that the improvement of human-machine interaction can leverage dynamic capability and reduce the inertia in enterprises through multiple nonlinear feedback circles. There are two paths to improving reliability and performance in enterprises: (1) human-machine interaction can promote initiative innovation (change) from endogenous power by improving dynamic capability, and (2) it can improve the transformation efficiency of knowledge and variation induced by exogenous environmental changes to improve the dynamic capability, thus to improve the flexibility and reliability.

Various practical implications are proposed in this research. There are two policies of digital transformation for managers to select to achieve the same effect, which include (1) focusing on one dimension of human-machine interaction (technical or cognitive interaction) significantly; (2) promoting both cognitive interaction and technical interaction to a medium level simultaneously; and (3) promoting users' feelings of social presence. This finding suggests that designing smart-manufacture should take into account anthropomorphic cues such as voices, characters, or feeling and body gestures may foster users' feeling of having an authentic social interaction with a smart object, especially when the technology mostly functions autonomously. In the direction of creating more intelligent enterprises, managers need to leverage the strengths of both humans and robots to produce superior judgments and productivity, so as to integrate both human creativity and digitally enabled capabilities ingeniously. For example, some small enterprises can prompt the knowledge transformation through the cognitive interaction of human-machine pairs to exploit the potential of existing digital equipment. Big enterprises can improve the technical interaction by digital investment to explore the potential of variation induced by the environmental changes.

Our current modelling efforts suffer from two main sets of limitations. Firstly, just as Larsen and Lomi's [24] works, we presented a “model of a model” as a “second-order” model which accentuates the demonstration of the theoretical narrative and assumptions, rather than a copy model of a specific enterprise. Secondly, the two dimensions of human-machine interaction are set as exogenous variables in this system. However, they are also the embedded components of this system which are affected by factors such as inertia accumulation, organizational structure, and investment strategies.

## Data Availability

The experimental data used to support the findings of this study are available from the corresponding author upon request.

## References

[1] Nardo M., Forino D., Murino T. (2020). The evolution of man-machine interaction: the role of human in Industry 4.0 paradigm. *Production & Manufacturing Research*.

[2] Frank A. G., Dalenogare L. S., Ayala N. F. (2019). Industry 4.0 technologies: implementation patterns in manufacturing companies. *International Journal of Production Economics*.

[3] Rzevski G. (1997). A framework for designing intelligent manufacturing systems. *Computers in Industry*.

[4] Frick W. (2015). When your boss wears metal pants. *Harvard Business Review*.

[5] Wesche J. S., Sonderegger A. (2019). When computers take the lead: the automation of leadership. *Computers in Human Behavior*.

[6] Miklósi Á, Korondi P., Matellán V., Gácsi M. (2017). Ethorobotics: a new approach to human-robot relationship. *Frontiers in Psychology*.

[7] Mori M. (1970). The uncanny valley. *Energy*.

[8] Fong T., Nourbakhsh I., Dautenhahn K. (2003). A survey of socially interactive robots. *Robotics and Autonomous Systems*.

[9] Breazeal C. (2003). Emotion and sociable humanoid robots. *International Journal of Human Computer Interaction*.

[10] Jarrahi M. H. (2018). Artificial intelligence and the future of work: human-AI symbiosis in organizational decision making. *Business Horizons*.

[11] Collins G. R. (2020). Improving human-robot interactions in hospitality settings. *International Hospitality Review*.

[12] Kulms P., Kopp S. (2018). A social cognition perspective on human-computer trust: the effect of perceived warmth and competence on trust in decision-making with computers. *Frontiers in Digital Humanities*.

[13] Schoemaker P., Tetlock P. E. (2017). Building a more intelligent enterprise. *MIT Sloan Management Review*.

[14] Card S., Moran T. P., Newell A. (1983). *The Psychology of Human-Computer Interaction*.

[15] Goodrich M. A., Schultz A. C. (2008). Human–robot interaction: a survey. *Foundations and Trends® in Human–Computer Interaction*.

[16] Morasso P. (2021). Gesture formation: a crucial building block for cognitive-based Human-Robot Partnership. *Cognitive Robotics*.

[17] Brondi S., Pivetti M., Battista S. D., Sarrica M. (2021). What do we expect from robots? Social representations, attitudes and evaluations of robots in daily life. *Technology in Society*.

[18] Pavlou P. A., El Sawy O. A. (2010). The “third hand”: IT-enabled competitive advantage in turbulence through improvisational capabilities. *Information Systems Research*.

[19] Pacaux-Lemoine M.-P., Trentesaux D., Rey G. R., Pattrik M. (2017). Designing intelligent manufacturing systems through Human-Machine cooperation principles: a human-centered approach. *Computers & Industrial Engineering*.

[20] Teece D. J., Pisano G., Shuen A. (1997). Dynamic capabilities and strategic management. *Strategic Management Journal*.

[21] Levitt B., March J. G. (1988). Organizational learning. *Annual Review of Sociology*.

[22] Wohlgemuth V., Wenzel M. (2016). Dynamic capabilities and routinization. *Journal of Business Research*.

[23] Gobet F., Sala G. (2019). How artificial intelligence can help us understand human creativity. *Frontiers in Psychology*.

[24] Larsen E., Lomi A. (2002). Representing change: a system model of organizational inertia and capabilities as dynamic accumulation processes. *Simulation Modelling Practice and Theory*.

[25] Kusiak A. (2018). Smart manufacturing. *International Journal of Production Research*.

[26] Sterman J. D. (2000). *Business Dynamics*.

[27] Bolisani E., Oltramari A. (2012). Knowledge as a measurable object in business contexts: a stock-and-flow approach. *Knowledge Management Research and Practice*.

